# Chicken Astrovirus (CAstV) Molecular Studies Reveal Evidence of Multiple Past Recombination Events in Sequences Originated from Clinical Samples of White Chick Syndrome (WCS) in Western Canada

**DOI:** 10.3390/v12101096

**Published:** 2020-09-28

**Authors:** Victor Palomino-Tapia, Darko Mitevski, Tom Inglis, Frank van der Meer, Emily Martin, Marina Brash, Chantale Provost, Carl A. Gagnon, Mohamed Faizal Abdul-Careem

**Affiliations:** 1 Department of Ecosystem and Public Health, Faculty of Veterinary Medicine, University of Calgary, Health Research Innovation Center 2C53, 3330 Hospital Drive NW, Calgary, AB T2N 4N1, Canada; victor.palominotapia@ucalgary.ca (V.P.-T.); fjvander@ucalgary.ca (F.v.d.M.); 2 Poultry Health Services, 1-4 East Lake Ave NE, Airdrie, AB T4A 2G8, Canada; darko.mitevski@poultryhealth.ca; 3 The Institute of Applied Poultry Technologies, 201–151 East Lake Blvd, Airdrie, AB T4A 2G1, Canada; tom.inglis@poultryhealth.ca; 4 Animal Health Laboratory, University of Guelph, Guelph, ON N1H 6R8, Canada; eamartin@uoguelph.ca (E.M.); mbrash@uoguelph.ca (M.B.); 5 Swine and poultry infectious diseases research center (CRIPA Fonds de Recherche du Quebec), Molecular Diagnostic Laboratory, Faculty of veterinary medicine, University of Montreal, 3200 Sicotte, Saint-Hyacinthe, QC J2S 2M2, Canada; chantale.provost@umontreal.ca (C.P.); carl.a.gagnon@umontreal.ca (C.A.G.)

**Keywords:** chicken astrovirus, white chick syndrome, runting-stunting syndrome, molecular epidemiology, whole genome sequencing, recombination

## Abstract

In this study, we aimed to molecularly characterize 14 whole genome sequences of chicken astrovirus (CAstV) isolated from samples obtained from white chick syndrome (WCS) outbreaks in Western Canada during the period of 2014–2019. Genome sequence comparisons showed all these sequences correspond to the novel Biv group from which no confirmed representatives were published in GenBank. Molecular recombination analyses using recombination detection software (i.e., RDP5 and SimPlot) and phylogenetic analyses suggest multiple past recombination events in open reading frame (ORF)1a, ORF1b, and ORF2. Our findings suggest that recombination events and the accumulation of point mutations may have contributed to the substantial genetic variation observed in CAstV and evidenced by the current seven antigenic sub-clusters hitherto described. This is the first paper that describes recombination events in CAstV following analysis of complete CAstV sequences originated in Canada.

## 1. Introduction

Chicken astrovirus (CAstV) [1], an enteric, non-enveloped, positive-sense RNA virus has recently emerged as an important poultry pathogen in broiler breeder flocks and their progeny across North America, Brazil, China, and several European countries including Poland, Finland, Norway, and United Kingdom [2,3,4,5,6,7,8,9]. Currently, the International Committee of Taxonomy of viruses (ICTV) in the latest 2019 edition has classified CAstV, together with avian nephritis virus (ANV), as members of the *Avastrovirus II* species within the genus *Avastrovirus*, in the Astroviridae family [10,11]. It is worth to note that classification of astroviruses has changed several times since first descriptions were published in the late 1970s to early 1980s [9,12,13]. The genetic organization of CAstV is similar to other astroviruses as it is composed of a small, linear RNA of ~7.5 kb in length, coding for three open reading frames (ORF): a non-structural protein (ORF1a), a viral RNA-dependent RNA polymerase (ORF1b, also named RdRp), and a capsid protein (ORF2) [3,5,14,15]. The capsid protein is highly variable, especially in its 3′ half of the ORF, which forms the external surface of the capsid forming the characteristically five or six-pointed star-like projections of astroviruses [16,17,18]. This area interacts with the cell receptor and is exposed to the host immune system [3,5,16]. The capsid protein has been divided into two major antigen groups with sub-divisions: Group A divided into three subgroups (i.e., Ai, Aii, and Aiii); and Group B divided into four subgroups (i.e., Bi, Bii, Biii, and Biv) [5].

Features of the capsid protein of CAstV are believed to drive the pathogenesis into three syndromes/diseases that are not mutually exclusive: (1) runting-stunting syndrome (RSS) characterized by malabsorption, enteritis, growth problems, and uneven flock performance [19]; (2) kidney disease and visceral gout characterized by high mortality in young broilers (up to 40%) [20]; and white chick hatchery disease or white chick syndrome (WCS), a disease characterized by transient increase in mid to late embryo deaths, which causes a reduction in hatchability that can be as low as 4–5% and as high as 68% [21]. In WCS, some of hatched chicks are considered “white chicks”, a condition characterized by pale plumage, weakness, slow weight gain, poor condition, and eventually death during the first days of life [3,6,21]. Lesions can be observed in kidney, liver, feathers, and intestine [3,5,6,21]. Although WCS has been known in Canada since the late 1980s–early 1990s, it has only recently been associated with CAstV in 2012 [4]. Improvements in surveillance and diagnostic assays have revealed an increased incidence of the problem, and its associated economic losses have rendered WCS a relevant emerging problem in poultry production in Canada [4,22,23].

Transmission of CAstV can be horizontal, through the fecal–oral route; and probably vertical, although this has not been experimentally proven [3,4,5]. In the case of WCS, the virus can be detected in dead-in-shell embryos, meconium, and young chickens within the first week of life [4,9,15]. Progenitor broiler breeders of affected broiler flocks usually have a history ranging from no hatchability decrease or a decrease of 68% [3,4,5]. Many studies agree that progenitor flocks, naïve to CAstV, are challenged during production, experience a variable decrease in hatchability (with birds hatching as “white chicks”), and return to normal parameters after ~4 week period where they become seropositive by commercial CAstV Group B ELISA testing [3,5,24].

In contrast to our knowledge of the molecular and epidemiologic characteristics of CAstV, our comprehension of CAstV pathogenesis is still scarce. Currently, the control of this disease is difficult due to its large geographical distribution, its horizontal, and likely vertical transmission, the environmental stability of the virus, and CAstV disinfection resistance. The lack of commercially-available vaccines may be in part due to the fact that CAstV is difficult to grow at immunogenic titers preventing cost-effective commercial vaccine production [3,4,25]. So far, the Canadian poultry industry is relying on strict biosecurity, increased down time between flocks, and effective disinfection of the premises. In some operations, the controversial practice of controlled-exposure by moving litter from CAstV ELISA-positive flocks into naïve pullet flocks is also used, despite the dangers of exposing naïve birds to other important pathogens such as *Mycoplasma* or *Salmonella* species.

Recently, several outbreaks of WCS were identified across Western Canada and Ontario. These outbreaks have caused sizable losses in Canadian poultry operations not only due to the detrimental effects of the disease, but also due to the sudden changes in allocation of day-old broiler chickens, which are of importance in the Canadian supply management system. We hypothesized that WCS cases detected since 2017 were associated with the presence of group B CAstV. Our objective was to characterize these CAstV isolates using Next Generation Sequencing (NGS) as a tool to study the genomic diversity of this virus in Western Canada.

## 2. Materials and Methods

### 2.1. Sample Collection, Histopathology, and Processing

Between December 2014–June 2019, a total of 17 samples from 12 clinical cases were diagnosed as WCS by Poultry Health Services (PHS, Airdrie, AB, Canada). Clinical samples, such as liver and intestines, were obtained from affected dead-in-shell embryos and young birds and tested by the Animal Health Laboratory (University of Guelph, Guelph, ON, Canada) for CAstV using quantitative polymerase chain reaction (qPCR) [23,26]. Affected tissues from some cases were also submitted to the same laboratory for histopathology examination for confirmation of diagnosis, and 17 samples from these clinical cases were held at −80 °C for further processing.

The liver and intestine samples were placed into sterile tubes prefilled with 1.0 mm zirconium beads (Benchmark Scientific Sayreville, NJ, USA), and 0.5 mL 1× phosphate buffered saline (PBS) (Gibco, Waltham, MA, USA) on ice, and homogenized (BeadBug, Benchmark Scientific, Sayreville, NJ, USA) during three series of 30 seconds each at 300 rounds per minute (RPM). Samples were incubated on ice for 3 minutes (min) in between series. Following disruption, the samples were centrifugated at 7500× *g* for 30 min at 4 °C and the supernatant filtered using a 0.2 µM syringe filter (Millipore Sigma, Burlington, MA, USA) and kept on ice for further processing.

### 2.2. Virus Propagation

Chicken embryo liver (CEL) cells were prepared using 14-day-old specific pathogen free (SPF) embryos obtained from the Canadian Food Inspection Agency (CFIA) (Ottawa, Canada). It has been shown previously that CEL can be infected with a variety of poultry viruses [27,28,29,30,31,32], including CAstV [1,33]. The use of embryos was approved by the institutional animal care committee, Health Science Animal Care Committee (HSACC). Livers were obtained from embryos following aseptic technique, minced, trypsinized (Gibco, Carlsbad, California, USA), and cultured in T25 flasks as previously described [29]. CELs were propagated in Dulbecco’s Modified Eagle’s Medium (DMEM) with 10% fetal bovine serum (FBS), and 100 U/mL penicillin, and 100 μg/mL streptomycin (Gibco, Carlsbad, California, USA). After viral infection, with processed samples (supernatants), similar media was used (except that 2% calf serum (CS) was used instead of 10% FBS). Cells were incubated at 37 °C with 5% CO_2_. Three passages in CEL were performed before ribonucleic acid (RNA)-extraction, complimentary deoxy ribonucleic acid (cDNA) conversion, and PCR for detection of CAstV by qPCR [23,26].

### 2.3. RNA Extraction, Reverse Transcription, qPCR, and Sequencing

Total RNA was extracted from infected CEL cultures supernatants, obtained after centrifugation at 7500× *g* for 30 min at 4 °C and the supernatant filtered using a 0.2 µM syringe filter (Millipore Sigma, Burlington, MA, USA), using TRIzol Reagent (Invitrogen, Carlsbad, CA), according to manufacturer’s instructions with modifications. In short, a total of ~1.5 mL of filtrated supernatant was pooled. The extracted RNA was used as template for reverse-transcription (RT) PCR using high-capacity cDNA reverse transcription kit (Applied Biosystems, Foster City, CA, USA) for cDNA synthesis using random primers following manufacturer’s instructions. The RT-PCR reaction mix consisted of 4 μL 10× RT Random Primers, 2 μL 10× RT Buffer, 4 mM dNTP Mix, 2 μL MultiScribe™ RT, 8.4 μL nuclease free H_2_O, and a 20 μL RNA template for a total of 40 μL reaction mix. RT-PCR thermocycler conditions consisted of three steps: Step 1-Incubation, 25 °C for 10 min; Step 2-Reverse Transcriptase, 37 °C for 120 min; and Step 3, Reverse transcriptase inactivation, 85 °C for 5 min. The qPCR assay was conducted using PerfeCTa SYBR Green SuperMix (Quantabio, Beverly, MA, USA) using cDNA as a template. The qPCR reaction mix was used using published primers by Smyth et al. 2010 [23] and consisted on 12.5 µL PerfeCTa SYBR 2× Buffer, 0.5 µM CAstV Forward Primer (5′-GCYGCTGCTGAAGAWATACAG-3′), 0.5 µM Reverse Primer (5′-CATCCCTCTACCAGATTTTCTGAAA-3′); 5 μL nuclease free H_2_O; and 5 μL cDNA template for a total of 25 μL reaction. The qPCR thermocycler conditions consisted of an initial denaturation cycle of 95 °C for 3 min, and 39 cycles of 95 °C for 15 seconds, 60 °C for 45 seconds. At the end of the amplification, a melting curve analysis was performed to verify the proper melting temperature of the amplicons. Conditions of the melt curve protocol consisted of 5 seconds at 65 °C and then 5 seconds each at 0.5 °C increments between 65 °C and 95 °C. Post-run qPCR amplification and melt-curve data were analyzed using the Bio-Rad CFX Maestro 1.1 software (v4.1.2433.1219) for positive identification of CAstV in the samples. Quantification (Nanodrop 1000, ThermoScientific, Wilmington DE, USA) of the cDNA was performed before submission for NGS using a Nextera XT library and the v3 600 cartridge (MiSeq, Illumina, San Diego, CA, USA) at the Faculty of Veterinary Medicine of University of Montreal, Montreal, QC, Canada. Prior to second strand synthesis, 1 µL RNAse H (New England Biolabs, MA, USA) was added to 20 µL cDNA to hydrolyze the RNA strand of the cDNA:RNA hybrid double strand, followed by a 20 min incubation at 37 °C. Then, 1 µL 60 µM Random Primers and 22 µL of nuclease free H_2_O were incubated for 5 min at 65 °C. Second strand synthesis was done using 5 µL 10× Buffer 2 and 1 µL Klenow Fragment (3′ → 5′ exo-) at 25 °C for 5 min, 37 °C for 50 min, and 75 °C for 15 min. Synthesized double stranded DNA (dsDNA) was cleaned with 1.8X Axygen AxyPrep Mag PCR Clean-up beads (Corning, NY, USA) following the manufacturer’s protocol. Quantification of dsDNA was performed using HS DNA Assay Kit in a Qubit 3.0 Fluorometer (Invitrogen, CA, USA). Libraries were generated using Nextera XT DNA Library Preparation Kit (Illumina, CA, USA). Briefly, 0.3 ng/mL of dsDNA was used to start the libraries. Fragmentation and tagmentation was performed as suggested by the company’s protocol. Amplification and indexing were also performed as described in the company’s protocol. Libraries were then purified using AxyPrep Mag™ PCR Clean-up Kits (Corning, NY, USA) as described in the Nextera XT protocol. Library’s quality was assessed using Agilent High Sensitivity DNA Kit in a Bioanalyzer (Agilent, CA, USA). Libraries were normalized using LNB1 beads (Nextera XT protocol). Libraries were sequenced in a v3 600 cartridge using a MiSeq instrument and PhiX at around 1% as control for the sequencing runs (Illumina, CA, USA).

### 2.4. Data Analysis and Phylogenetic Analysis

NGS short reads were mapped to the CAstV isolate CkP5 (GenBank accession# KX397576), under App Map function of CLC Genomics Workbench v 12.0.2 (Qiagen, Valencia, CA, USA) using default settings. Whole genome sequences were aligned using MAFFT v7.450 [34,35], and phylogenetic trees were generated using RAxML v8.2.11. This was possible by applying the nucleotide model GTR+gamma with Rapid bootstrapping and searching for best-scoring ML tree with 1000 bootstrap replicates, with parsimony random seed 400,000 as in previous studies concerning other RNA-viruses [36,37,38]. It is worth to mention that selection of this model over others was done based on its high frequency in similar studies, albeit it is expected that different models will lead to very similar results according to Abadi, et al. 2019 [38]. ORF1a, ORF1b, and ORF2 nucleotide and amino acid (aa) alignments were performed using Clustal Omega v1.2.2. and phylogenetic trees were generated using RAxML applying the protein model BLOSUM62+gamma with Rapid bootstrapping and search for best-scoring ML tree with 1000 bootstrap replicates, with parsimony random seed 400,000. All the sequences were deposited in GenBank (Table 1). For phylogenetic analysis on ORF2, 38 aa sequences from CAstV reference strains and field sequences from different locations around the world were retrieved from GenBank and included in the study (Table 2) [4,5]. The CAstV classification based on ORF2 aa sequence was based on two criteria: (1) Bootstrap values of the RAxML phylogenetic trees with 1000 replicates, and (2) percentage of identity matrix resulting from the RAxML phylogenetic tree as in a previous publication based on a different virus, avian reovirus [37].

### 2.5. Recombination Analysis

To identify the presence of recombinant sequences, a multiple sequence alignment was performed including all 24 complete CAstV sequences using MAFFT v7.450 [34,35] (Table 2). The sequence analysis was analyzed in RDP5 software v. 5.5 [39,40,41], which is a software that applies several recombination and analysis methods on a set of data. In this research, data was analyzed using the following recombination methods: (1) RDP method [40]; (2) GENECONV [42]; (3) Bootscan/Recscan method [39]; (4) MaxChi method [43]; (5) Chimaera method [44]; (6) SiScan Method [45]; and (7) 3-seq [46]. Recombination events were detected by at least 6 of these 7 methods. Putative recombination sequences were further investigated by using Bootscan analysis within the Simplot program version 3.5.1 [47], using the following parameters: window size = 400 bp; step size = 40 bp; GapStrip = On; Repetitions = 100; Kimura 2-parameter substitution model; T/t = 2; and using the neighbor-joining method [47,48,49].

## 3. Results

### 3.1. Clinical Background, Gross Lesions, and Histopathology

**Figure 1:**
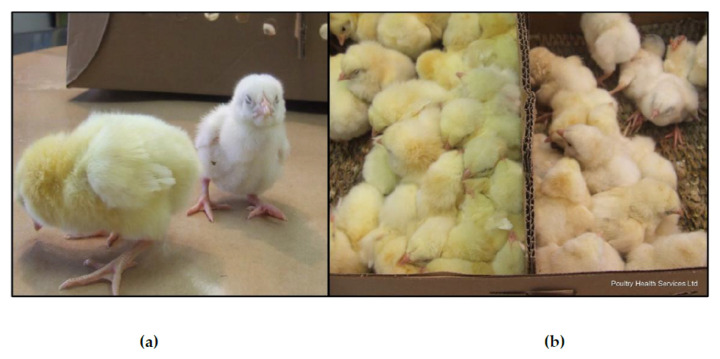
Hatching of normal (yellow) and affected (white) chicks. Progenitor broiler breeders had a drop in production, and low hatchability (case 14-1235). Apparently normal chick on the left and an affected “white chick” on the right in (**a**). Chick box after quality check containing apparently normal chicks on the left, and affected chicks on the right (**b**).

**Figure 2:**
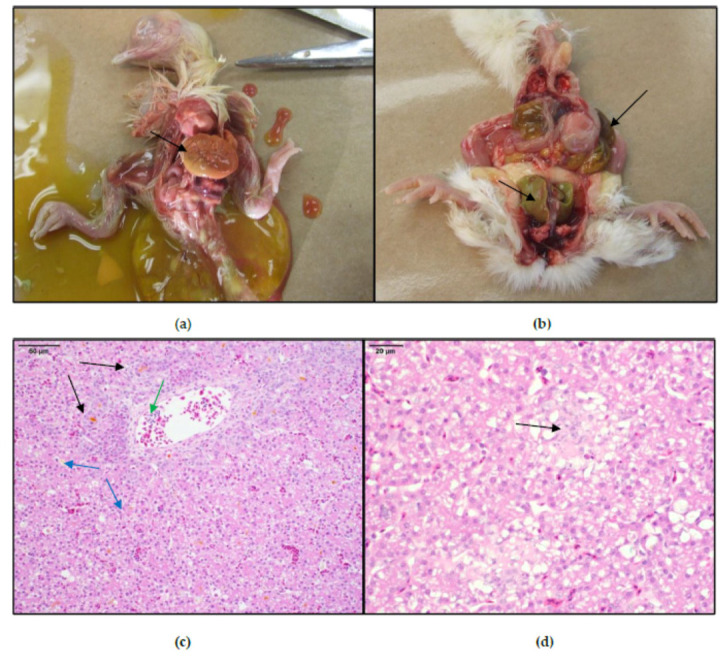
Post-mortem examination on dead-in-shell embryos and culls of case 15-1262a, and histopathology of cases 14-1235a; and 15-1262a, respectively. Dead-in-shell embryo with enlarged firm green livers in (**a**). Day-old culled chick with white plumage showing enlarged firm green liver and unabsorbed yolk-sac contents with visible green discoloration in (**b**). CAstV microphotographs of histopathological liver lesions on white chick syndrome (WCS) clinical cases. Case 14-1235a shows in 20× (**c**), proliferating bile ducts in black arrows, bile in canalicular lumens in blue arrows, and heterophils and macrophages in the portal vain in the green arrow. Case 15-1262a (**d**) shows in 40× one small foci of acute hepatic necrosis (black arrow).

The hatching of WCS-affected broilers on the diagnosed clinical cases was characterized by low uniformity, increased culls with green livers and white feathering (Figure 1 and Figure 2d). Records of hatchability losses, when available, ranged between 5 and 16%, and occurred mainly in the mid and late incubation periods (Table 1). Dead-in-shell embryos were characterized by enlarged firm livers ranging from bronze to bright green with occasional necrotic areas with large, underutilized, and unabsorbed yolk-sac contents with visible green discoloration (Figure 2a,b). Some embryos were found covered in what would appear to be urates. Cull chicks were characterized by small size, depression, weak upon stimulation, and frequently white plumage (Figure 1a). Upon necropsy, green livers, and dark-green unabsorbed yolks were observed in these birds (Figure 2b). In some instances (17-0823), high mortality was observed during the first week of life in flocks apparently mildly affected with WCS during hatching. In this case, 6 day of age (DOA) were submitted with a history of high first week mortality of about 0.25% per day, RSS, and swollen/pale kidneys and mottled livers together with yolk sac infection. The age of submission of the cases included in this study was between 18 DOE, and the latest at 6 DOA, with a median of 1 DOA. These cases came from broiler breeder flocks between 28 and 40 weeks of age with most of the cases occurring from progenitors at the beginning of the production cycle, between 28 and 33 weeks. Clinical necropsies and sample collections from submitted cases were performed by veterinarians and trained PHS personnel at the post-mortem facility at the Veterinary Professional Center (Airdrie, AB, Canada), following guidelines approved by the Alberta Veterinary Medical Association, the CFIA, and the Public Health Agency of Canada (PHAC).

In 14 of the 17 samples tested, we were able to isolate CAstV in CEL (Table 1). We speculate that the other three samples were not able to be isolated in CEL due to low amount of initial virus (high Ct value) or lack of viable virus.

Under microscopic examination of livers obtained from clinical cases (Figure 2c,d), affected livers showed mild to severe biliary proliferation with periportal stores of immature granulocytes. Bile ducts usually lined with hyperplastic epithelium and many are dilated, containing necrotic heterophils and eosinophilic debris. It is common to find acute peribiliary inflammation with accumulation of necrotic heterophils and accumulations of orange/eosinophilic fluid compatible with bile in the bile ducts and surrounding tissues (Figure 2c—black arrows). The same fluid can be found pooling in canaliculi (Figure 2c—blue arrows), with variably sized foci of acute periportal hepatic necrosis with mild hemorrhage and small periportal aggregates of immature heterophils. It is thought that this trapped bile is the responsible of the characteristic green color of some affected livers seen in WCS cases.

### 3.2. Whole Genome Sequencing

**Figure 3:**
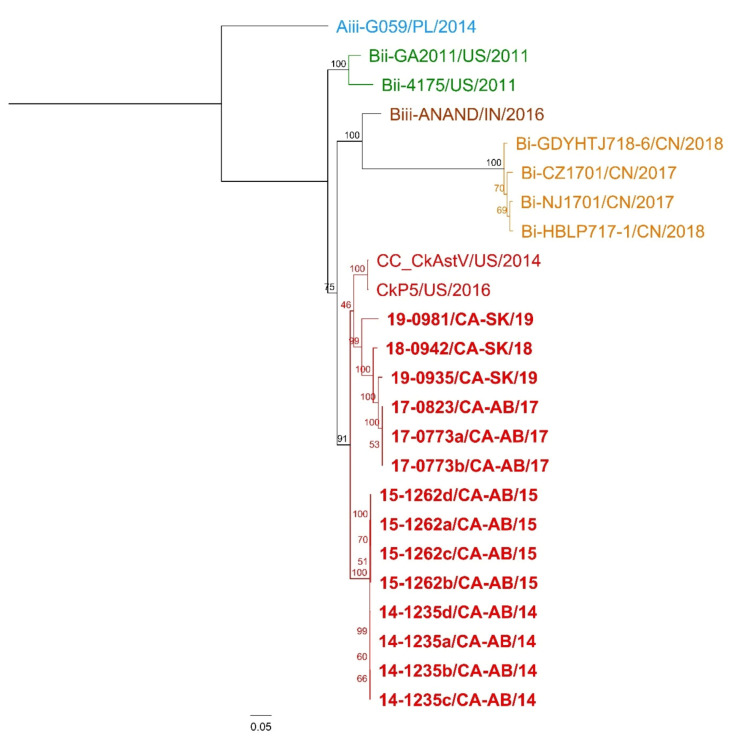
Nucleotide ML phylogenetic tree of complete CAstV sequences. Different colors indicate different genotypes according to ORF2 analysis described in Smyth et al. 2017 (i.e., Aiii, Bi, Bii, Biii, and Biv in red) [5]. The included sequences are described in Supplementary Table S1. Canadian sequences are in bold.

**Figure 4:**
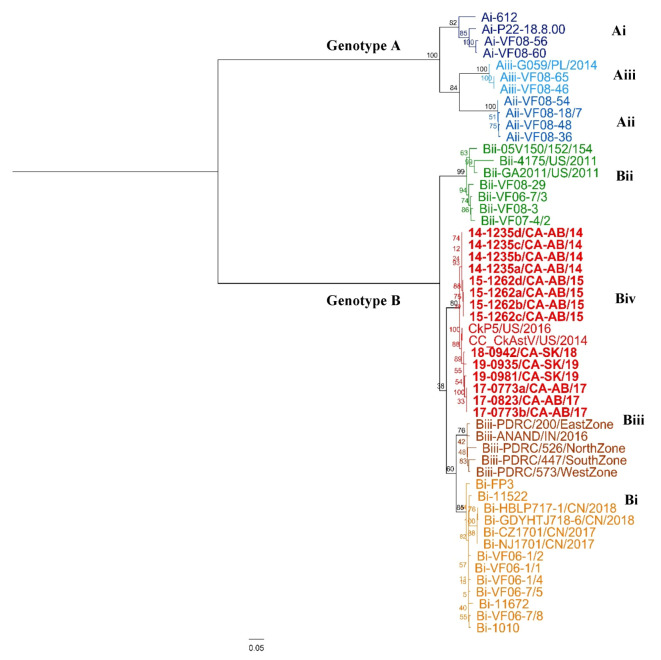
Amino acid ML phylogenetic tree of 52 ORF2 CAstV sequences. Different colors indicate different genotypes according to ORF2 analysis described by Smyth 2017 [5]. The included sequences are described in Supplementary Table S1.

The complete genome sequences of CAstV isolates (*n* = 14) with their corresponding genome size are shown in Supplementary Table S1. Other complete sequences (*n* = 10) were included in the analysis and genotype, phylogenetic tree constructed, and publication from which they were obtained are shown in Table 2. For all samples, the full coding sequence was obtained, and most of the 5′ and 3′ non-coding regions were also obtained by Bioinformatic resequencing analysis performed with CLC Genomics Workbench v 12.0.2 (Qiagen, Valencia, CA, USA). In the present study, whole genome phylogenetic analysis was performed on 24 complete CAstV sequences, showed that all 14 CAstV sequences circulating in Western Canada clustered with CAstV from United States (US): CC_CkAstV/US/2014, and CkP5/US/2016. The same clustering corresponded to their ORF2 genotype (Figure 3). All sequences analyzed in this study were included in a separate cluster within Genotype B, different from Aiii (G059/PL/2014); Bi (Chinese strains: CZ1701/CN/2017; HBLP717-1/CN/2018; NJ1701/CN/2017; and GDYHTJ718-6/CN/2018); Bii (US strains: GA2011/US/2011; 4175/US/2011); Biii (Indian strain: ANAND/IN/2016); and in the same cluster as US strains CkP5/US/2016; and CC_CkAstV/US/2014. To our knowledge, no complete CAstV genome characterized as genotype Biv has been uploaded to GenBank. The following findings were observed in the whole genome alignment: (1) Consensus sequence with 7809 nucleotides (nt), with ungapped lengths of 24 sequences: Mean = 7479.6 nt, Minimum = 7008 nt, Maximum = 7603 nt, Std Dev = 108.45 nt; (2) 3,879 nt identical sites with 3930 nt (50%); (3) 86.1% nt pairwise identity; and (4) 204 nt gaps with 43 of those gaps in coding sequences (ORF1a, ORF1b, and ORF2). These changes resulted into 917 non-synonymous mutations in ORF1a, ORF1b, and ORF2. Phylogenetic trees (Figure 3 and Figure 4) showed Nucleotide RAxML phylogenetic tree of complete CAstV sequences clustered in a similar way as the aa RaxML based phylogenetic tree of ORF2 CAstV sequences, but these two phylogenetic trees (Figure 3 and Figure 4) clustered differently as aa RAxML phylogenetic trees of ORF1a, and ORF1b (Supplement Figures S1 and S2).

### 3.3. ORF1a

A total of 1274 nt mutations, and 9 nt gaps were identified in the ORF1a gene, rendering 246 aa changes and 3 gaps in a consensus sequence of 1142 aa (21.5%) out of 24 sequences (Supplementary Table S1) [2,15,50,51]. Out of the three coding regions in CAstV, ORF1a was the one with the lowest aa variation. Many of the non-synonymous mutations present were specific to genotypes A or B; thus, the ORF1a phylogenetic tree in Supplementary Figure S1, shows that genotypes A and B clustered separately. However, unlike Figure 3 (whole genome phylogenetic tree), and Figure 4 (ORF2 phylogenetic tree), not all sequences within ORF2 genotype B clustered according to their sub-genotype. For instance, sequence Bii-4175/US/2011 clustered near Biv sequences CkP5/US/2016, and CC_CkAst/US/2014; and sequences Bii-GA2011/US/2011 and Biii-ANAND/IN/2016 close to Canadian isolates obtained in this research during 2014, and 2015 (Supplementary Figure S1).

### 3.4. ORF1b

A total of 1563 nt mutations, and 3 nt gaps were identified in the ORF1b gene, rendering 139 aa changes, and 3 aa gaps in a consensus sequence of 521aa (26.7%) out of 24 sequences (Supplementary Table S1) [2,15,50,51]. The phylogenetic tree in Supplementary Figure S2 shows that genotypes A and B clustered separately. However, unlike Figure 3 (whole genome phylogenetic tree), and Figure 4 (ORF2 phylogenetic tree), and similarly to Supplementary Figure S1 (ORF1a phylogenetic tree) not all sequences within ORF2 genotype B clustered according to their sub-genotype. For instance, sequence Bii-4175/US/2011 clustered in between Canadian CAstV isolated in 2014/2015 and 2017/2018/2019; and Bii-GA2011/US/2011 clustered near Biv sequences CkP5/US/2016, and CC_CkAst/US/2014. This, in contrast with ORF1a tree (Supplementary Figure S1), and Biii-ANAND/IN/2016 close to Canadian isolates obtained in 2014, and 2015 (Supplementary Figure S1).

### 3.5. Genotyping and Comparison of ORF2

Comparison between all previously published ORF2 sequences in research papers (*n* = 14) (Table 2) [2,5,15,50,51], GenBank, and those obtained in the current work (*n* = 38; 52 sequences in total), showed the presence of several unique and shared mutations in 1728 nt in a consensus sequence of 2267 nt (76.2%) with 49 nt gaps in a consensus sequence. These mutations translated into 591 aa non-synonymous mutations in a 788 aa-length with 30 aa gaps in a consensus protein sequence. Out of the three coding regions in CAstV, ORF2 was the one with the highest aa variation.

The 14 Western Canadian isolates together with CkP5/US/2016 and CC_CkAstV/US/2014 clustered into a subgroup different from all published reference strains analyzed (Figure 4). As no representative sequence genotyped as Biv CAstV antigenic group was uploaded to GenBank, the authors contacted Dr. Victoria Smyth from the Agri-Food and Bioscience Institute in Belfast, United Kingdom, who confirmed that reference strains CkP5/US/2016, CC_CkAst/US/2014, and 14-1235a were classified within the Biv subcluster and share 97.8–98.8% aa similarity with VF11-71, a Canadian isolate obtained from a case of WCS, and 95.0–96.7% aa similarity with WCS European sequences VF10-26, and VF11-66 [5,52]. Amino acid sequence identity between Western Canada sequences was of 96.88–100%; and when compared with US sequences corresponding to Biv group, aa sequence identity varied from 97.97 to 98.51%. Other aa sequence identities within groups were: Group A—75.83–100%: Ai—89.72–99.03%; Aii—99.03–99.45%; Aiii—98.33–100%; Group B—77.05–100%; Bi—95.66–100%; Bii—88.43–98.79%; Biii—94.99–98.37%; and Biv—96.88–100%. Groups A and B were dissimilar and only shared 33.53–38.93% of aa identity.

### 3.6. Recombination Analysis

There was a total of 36 recombination events found by RDP5 software using the full exploratory recombination scan function, but only 12 were supported by at least 6 of 7 algorithms, as indicated in the Material and Methods section on 24 complete CAstV genomes. Table 3 shows a summary of those 12 events, the recombinant and major parent (P1) and minor parent (P2), the number of recombination methods supporting the events, *p*-value ranges, and most likely position of breaking points.

**Figure 5:**
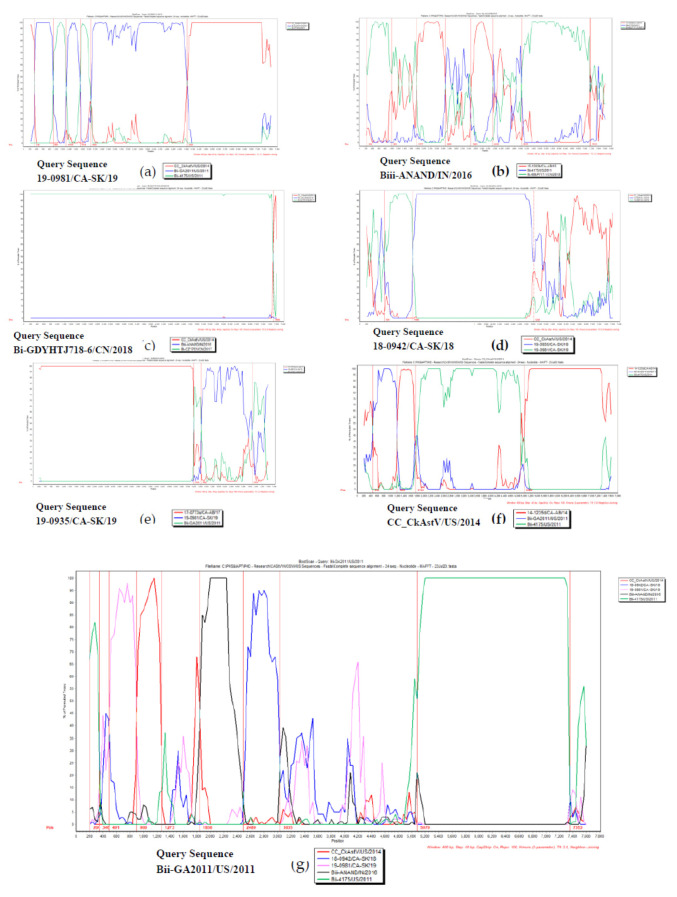
Bootscan analysis of recombinant CAstV sequences for confirming recombination was performed using Simplot program v3.5.1. Each analysis considers different parent sequences (different colors) plotted in a graph considering in the vertical-axis Percentage of permuted trees, and on the horizontal axis, position on the genome of the query sequence. Recombinant CAstV query sequences are 19-0981/CA-SK/19 (Supplementary Figure S3a); Biii-ANAND/IN/2016 (Supplementary Figure S3b); Bi-GDYHTJ718-6/CN/2018 (Supplementary Figure S3c); 18-0942/CA-SK/18 (Supplementary Figure S3d); 19-0935/CA-SK/19 (Supplementary Figure S3e); CC_CkAstV/US/2014 (Supplementary Figure S3f); and Bii-GA2011/US/2011 (Supplementary Figure S3g).

In addition, ML phylogenetic trees were generated based on each event breakpoints to evidence relations between recombinant and parental sequences (Supplementary Figure S3). Putative recombinant sequences Biv-19-0981/CA-SK/19; Biii-ANAND/IN/2016; Bi-GDYHTJ718-6/CN/2018; Biv-18-0942/CA-SK/18; Biv-19-0935/CA-SK/19; Biv-CC_CkAstV/US/2014; and Bii-GA2011/US/2011 were further analyzed using the Bootscan analysis within the Simplot program (Figure 5). Based on all these three previous analyses, the evidence suggests the presence of seven recombinant sequences (Table 4).

## 4. Discussion

For the last 30 years, WCS has been increasingly gaining relevance in North America and Europe [5,21,53,54,55,56]. Thus, it is crucial to understand the antigenic variation of the field challenge in our production systems in order to implement better control strategies. The objective of the present study was to molecularly characterize complete sequences of CAstV isolates causing WCS in Western Canada since 2014. The tissue culture level of passage of the isolates used in this study was only of three passages, which lowers the possibility of genetic adaptations to in vitro system and not originally found in the isolate. This is supported by one study and two regulatory agencies. The study, published in 2008, examined the highly variable S protein of infectious bronchitis virus (IBV), in 8 strains for up to 10 passages in vitro (egg passage); in this study, six strains had no changes, two had two non-synonymous changes, and only one had non-synonymous change [57]. Furthermore, regulatory agencies in Europe [58] and US [59] consider that the production of a licensed commercial vaccine should occur within five passages from the virus master seed as to warrant preservation of master seed characteristics based on internal testing. This is specified in case of the US regulation (9CFR) for the following poultry viral vaccines: avian encephalomyelitis, avian pox, infectious bronchitis, infectious laryngotracheitis, Newcastle disease, infectious bursal disease, and avian reo viral arthritis [59].

All 14 CAstV sequences circulating in Western Canada clustered together with CAstV from the US: CC_CkAstV/US/2014, and CkP5/US/2016 in a sub-cluster within the B genotype. Interestingly, these US sequences are linked not to a WCS case but to an RSS case in broilers in the US, in which CC_CkAstV/Us/2014 corresponds to the isolation in Leghorn Male Hepathoma (LMH) cells and CkP5/US/2016 to the fifth passage of this parent virus in chickens. This finding provides circumstantial evidence suggesting that some Canadian CAstV isolates obtained from WCS may have a RSS phenotype as well, which would have to be confirmed by animal studies. The genome organization (ORF1a, ORF1b, and ORF2) in the sequences described in this work were similar to the ones described previously [2,15,50,51]. Further classification of ORF2 coding sequence showed that, in agreement with the complete genome sequence analysis—but not with ORF1a, and ORF1b phylogenetic trees—all these sequences grouped together in a cluster within group B but independent from subgroups Bi, Bii, and Biii. Dr. Victoria Smyth from the Agri-Food and Bioscience Institute in Belfast, United Kingdom, kindly confirmed the location of representative strains within the subcluster Biv [5,52]. Thus, we considered all these Canadian sequences to be part of the subcluster Biv of CAstV, in agreement with other literature describing WCS phenotype hitherto in genotypes Biv and Aiii.

Other CAstV were detected circulating in Ontario, Canada, in recent years by Long et al. [4,22]. The 31 sequences described by Long et al. 2018 were originally classified as Bii group; however, this classification was based using only a partial ORF2 sequence consisting of 644 nt of a ~2269 nt ORF2 consensus, which represents a ~29% coverage. After analyzing the sequences from this study adjusted to ~644 nt, many of the sequences classified as Biv and Biii groups, were classified as Bii (Supplementary Figures S4 and S5). Thus, it is possible that these Canadian sequences originally classified as Bii using a partial sequence analysis, would be classified as Biv or Biii genotypes when the entire ORF2 gene is analyzed. Furthermore, the authors identify that this issue poses a major risk for epidemiology: due to its high variability, laboratory identification needs to be uniform and rely on the analysis of the entire CAstV ORF2 gene prior to classification lest misclassify relevant outbreaks. This is particularly relevant when designing an autogenous vaccine program to contain such outbreaks.

Upon analyzing the data more closely, we observed that the ORF1a and ORF1b phylogenetic trees did not follow the cluster pattern observed on the whole genome and capsid protein (ORF2) phylogenetic trees, which is suggestive of a recombination event. Upon analyzing the sequences for recombination, we found 12 novel recombination events between viruses of the Genotype B groups, which suggests cocirculation of these different viruses in each point in their evolution. One third (*n* = 4, Events 7, 8, 9, and 10) of these events occurred between members of the same subcluster (Biv) producing a recombinant from the same subcluster Biv; while only one (*n* = 1, Event 15) produced a recombinant classified as a different subcluster (Bii). One half of these events (*n* = 6, Events 3, 4, 5, 6, 12, and 13) involved one parent from a Biv group and another from Bii group, producing a variety of recombinant sequences (3 Biv, 1 Biii, 1 Bi, and 1 Biv). Finally, one last event (*n* = 1, Event 14), considered one parent from subgroup Biv and another from Biii, producing a Bii recombinant.

Recombination events and mutations (i.e., large-scale, and small-scale) [60] are the main drivers of evolution in RNA viruses, and such events need to be considered and studied [61,62,63,64]. Although mutations can be analyzed by phylogenetic trees and used for tracking the spread of a virus sequence, these trees are built under the assumption of no recombination [65,66]. Thus, observations of high phylogenetic diversity, such as the one found in the present work, have been suggestive or indicative of recombination events [67,68]. Astrovirus recombination events have been described in poultry such as turkeys [69,70], ducks [71], and guinea fowl [72]. To the best of our knowledge, no recombination event has been described in chickens, albeit interspecies recombination has been suggested [50,51]. Events between astroviruses from the same species [73,74,75] or, more rarely, from different species, may cause a change in host or tissue tropism [76,77,78], and are more difficult to study when only partial genomes are collected, as many of the recombination events do not occur solely in the areas of genome that represent antigenic proteins [79]. Research in CAstV has been focused more on partial or complete analysis of coding sequences, as currently there are 310 CAstV sequences available in GenBank, from which only 115 (~37%) correspond to complete or partial ORF2, 184 (~59%) to partial ORF1b, and only 11 (~3.5%) correspond to whole genome sequences.

## 5. Conclusions

In the present study, we isolated and sequenced 14 CAstV sequences from WCS cases. These were genotyped and classified within the novel Biv sub-cluster of CAstV, according to the ORF2 (capsid) genotypic classification. The molecular characterization and phylogenetic studies suggested multiple past recombination events with several CAstV sequences, some of them from US origin, linked to RSS-cases. Our findings suggest that recombination events and the accumulation of point mutations may have contributed to the great genetic variation observed in CAstV and evidenced by the current seven antigenic sub-clusters described above. This is the first paper that describes recombination events in CAstV following analysis of complete CAstV sequences originated in Canada. Based on the information presented in this paper, whole genome sequencing methods are also a powerful and useful tool that allows better characterization of the CAstV strains circulating in the field.

## Figures and Tables

**Table 1:** List and classification of 14 CAstV isolates deposited in GenBank and background information.

#	CAstV ID e	Capsid Genotyping	Origin	Province	Breeder Age	Age	Clinical Case	GenBank Accession
1	14-1235 a	Biv	Liver	AB	30W	1 DOA b	Flock A. Drop in production, very poor hatchability and poor viability of hatched chicks.	MT789774
2	14-1235 b	Biv	Intestine	AB	30W	1 DOA		MT789775
3	14-1235 c	Biv	Intestine	AB	28W	1 DOA	Flock B. Drop in production, very poor hatchability and poor viability of hatched chicks.	MT789776
4	14-1235 d	Biv	Liver	AB	28W	1 DOA		MT789777
5	15-1262 a	Biv	Liver	AB	32W	1 DOA	Flock A. Poor hatchability. Slow hatching eggs. Red hocks on many chicks, yellow livers. No white chicks.	MT789778
6	15-1262 b	Biv	Liver	AB	32W	1 DOA		MT789779
7	15-1262 c	Biv	Liver	AB	33W	1 DOA	Flock B. Poor hatchability. Slow hatching eggs. Increased culls with green livers and white chicks.	MT789780
8	15-1262 d	Biv	Liver	AB	33W	1 DOA		MT789781
9	17-0773 a	Biv	Liver	AB	30W	~20 DOE c	Flock A. Poor hatchability. Increased culls were weak with green livers and white feathering.	MT789782
10	17-0773 b	Biv	Liver	AB	30W	1 DOA		MT789783
11	17-0823	Biv	Liver	AB	NDa	6 DOA	High first week mortality at 0.25% per day- RSS d with swollen/pale kidneys and mottled livers.	MT789784
12	18-0942	Biv	Liver	SK	40W	1 DOA	Fertility 92%; hatchability 79%. High number of culls, 70% of them small and white with bronze/tan livers.	MT789785
13	19-0935	Biv	Liver	SK	28W	1 DOA	Fertility 81%; hatchability 68.9%; Culls 2– 90% of culls were white.	MT789786
14	19-0981	Biv	Liver	SK	38W	1 DOA	Fertility 92.2%; hatchability 84.3%; Culls 1.42%—25–30% culls are white.	MT789787

^a^ ND—No Data. ^b^ DOA—Days of Age. ^c^ DOE—Days of Embryonation. ^d^ RSS—Runting-Stunting Syndrome. ^e^ Number on name of CAstV ID correspond to clinical case. Some clinical cases were created by Hatchery. Thus, the letters following the number are used to differentiate some isolates in regards of Farm, organ, or age of bird/embryo.

**Table 2:** List of all CAstV sequences in the study with GenBank Accession Numbers.

Sequence	Genotype	Phylogenetic Tree				GenBank Number	Paper Published
		Whole Genome	ORF1a	ORF1b	ORF2		
14-1235a-AB	Biv	X	X	X	X	MT789774	This Study
14-1235b-AB	Biv	X	X	X	X	MT789775	
14-1235c-AB	Biv	X	X	X	X	MT789776	
14-1235d-AB	Biv	X	X	X	X	MT789777	
15-1262a-AB	Biv	X	X	X	X	MT789778	
15-1262b-AB	Biv	X	X	X	X	MT789779	
15-1262c-AB	Biv	X	X	X	X	MT789780	
15-1262d-AB	Biv	X	X	X	X	MT789781	
17-0773a-AB	Biv	X	X	X	X	MT789782	
17-0773b-AB	Biv	X	X	X	X	MT789783	
17-0823-AB	Biv	X	X	X	X	MT789784	
18-0942-SK	Biv	X	X	X	X	MT789785	
19-0935-SK	Biv	X	X	X	X	MT789786	
19-0981-SK	Biv	X	X	X	X	MT789787	
HBLP717-1/CN/2018 *	Bi	X	X	X	X	MN725025	[2]
GDYHTJ718-6/CN/2018 *	Bi	X	X	X	X	MN725026	
GA2011/US/2011 **	Bii	X	X	X	X	JF414802	[15]
CkP5/US/2016 **	Biv	X	X	X	X	KX397576	
CC_CkAstV/US/2014 **	Biv	X	X	X	X	KX397575	
ANAND/IN/2016 ***	Biii	X	X	X	X	KY038163	[50]
G059/PL/2014 ****	Aiii	X	X	X	X	KT886453	[51]
4175/US/2011 **	Bii	X	X	X	X	JF832365	Unpublished, 2011
CZ1701/CN/2017 *	Bi	X	X	X	X	MN807051	Unpublished, 2019
NJ1701/CN/2017 *	Bi	X	X	X	X	MK746105	
612	Ai				X	JN582317	[5]
P22-18.8.00	Ai				X	JN582318	
VF08-56	Ai				X	JN582319	
VF08-60	Ai				X	JN582320	
VF08-54	Aii				X	JN582323	
VF08-18/7	Aii				X	JN582324	
VF08-36	Aii				X	JN582325	
VF08-48	Aii				X	JN582326	
VF08-46	Aiii				X	JN582321	
VF08-65	Aiii				X	JN582322	
1010	Bi				X	JN582306	
11522	Bi				X	JN582305	
11672	Bi				X	JN582327	
FP3	Bi				X	JN582328	
VF06-1/1	Bi				X	JN582307	
VF06-1/2	Bi				X	JN582308	
VF06-1/4	Bi				X	JN582309	
VF06-7/5	Bi				X	JN582310	
VF06-7/8	Bi				X	JN582311	
05V150/152/154	Bii				X	JN582312	
VF06-7/3	Bii				X	JN582313	
VF07-4/2	Bii				X	JN582314	
VF08-29	Bii				X	JN582315	
VF08-3	Bii				X	JN582316	
PDRC/200/EastZone	Biii				X	JX945853	Unpublished, 2013
PDRC/526/NorthZone	Biii				X	JX945857	
PDRC/573/WestZone	Biii				X	JX945861	
PDRC/447/SouthZone	Biii				X	KC618323	

* CN refers to China as origin of the sequence; ** US refers to United States as the origin of the sequence; *** IN refers to India as the origin of the sequence; **** PL refers to Poland as the origin of the sequence.

**Table 3:** Details on Recombination Events detected by at least 6 methods on alignment of 24-CAstV complete sequences.

Event	Recombinant (R)& Parents (P1, P2)	No. of Methods	*P*-Value Range	Position of Breaking Points
3	(R)- 19-0981/CA-SK/19 P1- Bii-GA2011/US/2011 P2- 19-0935/CA-SK/19	6	1.811 × 10^−28^–1.695 × 10^−90^	ORF2 Start: 5090 nt End: 7750 nt
4	(R)- 19-0981/CA-SK/19 P1- Bii-4175/US/2011 P2- 19-0935/CA-SK/19	6	5.440 × 10^−11^–8.389 × 10^−86^	ORF2 Start: 5088 nt End: 84 nt
5	(R)- Biii-ANAND/IN/2016 P1- Bi-HBLP717-1/CN/2018 P2- 15-1262b/CA-AB/15	7	7.876 × 10^−06^–1.566 × 10^−86^	ORf2 Start: 7770 nt End: 5470 nt
6	(R)- Bi-GDYHTJ718-6/CN/2018 P1- Bi-CZ1701/CN/2017 P2- CC_CkAstV/US/2014	6	1.501 × 10^−07^–2.149 × 10^−32^	Start: 7606 nt End: 130 nt
7	(R)- 18-0942/CA-SK/18 P1- 19-0981/CA-SK/19 P2- 19-0935/CA-SK/19	7	5.222 × 10^−11^–4.321 × 10^−46^ *	ORF1a-ORF1b Start: 1507 nt End: 5332 nt
8	(R)- 18-0942/CA-SK/18 P1- Bi-GDYHTJ718-6/CN/2018 P2- Bii-GA2011/US/2011	6	1.613 × 10^−04^–1.910 × 10^−14^ **	ORF2 Start: ~7294 nt End: 5620 nt
9	(R)- 19-0935/CA-SK/19 P1- 17-0773a/CA-AB/17 P2- 19-0981/CA-SK/19	6	1.129 × 10^−03^–1.876 × 10^−13^	ORF2 Start: 5098 nt End: 7573 nt
10	(R)- 19-0981/CA-SK/19 P1- 15-1262b/CA-AB/15 P2- 17-0773a/CA-AB/17	6	3.210 × 10^−07^–1.015 × 10^−23^	ORF2 Start: 5133 nt End: 804 nt
12	(R)- CC_CkAstV/US/2014 P1- 14-1235d/CA-AB/14 P2- Bii-4175/US/2011	6	1.171 × 10^−05^–3.033 × 10^−23^	ORF1a-ORF1b Start: 2818 nt End: ~4929 nt
13	(R)- Bii-GA2011/US/2011 P1- Bii-4175/US/2011 P2- 18-0942/CA-SK/18	6	1.630 × 10^−02^–3.383 × 10^−09^	ORF1a-ORF1b Start: 2408 nt End: 4012 nt
14	(R)- Bii-GA2011/US/2011 P1- 18-0942/CA-SK/18 P2- Biii-ANAND/IN/2016	7	1.088 × 10^−03^–9.808 × 10^−08^	ORF1a Start: 1912 nt End: ~2407 nt
15	(R)- Bii-GA2011/US/2011 P1- 19-0981/CA-SK/19 P2- CC_CkAstV/US/2014	7	1.167 × 10^−03^–3.633 × 10^−08^	ORF1a Start: 1000 nt End: 1327 nt

* Beginning breakpoint outside of confidence interval. ** Recombination signal may be attributable to a process other than recombination. ~Unknown breaking point, approximate location noted.

**Table 4:** CAstV recombinant sequences and parents/parent-like sequences detected by 6 recombination methods in RDP5, ML phylogenetic trees, and Bootscan analysis in SimPlot software.

#	Recombinant Genotype	Recombinant Sequence	Parental Genotype	Parent Sequences
1	Biv	19-0981/CA-SK/19	Biv	CC_CkAstV/US/2014
			Bii	GA2011/US/2011
			Bii	4175/US/2011
2	Biii	ANAND/IN/2016	Bi	HBLP717-1/CN/2018
			Biv	15-1262b/CA-AB/15
3	Bi	GDYHTJ718-6/CN/2018	Bi	CZ1701/CN/2017
			Biv	CC_CkAstV/US/2014
4	Biv	18-0942/CA-SK/18	Biv	19-0981/CA-SK/19
			Biv	19-0935/CA-SK/19
5	Biv	Biv-19-0935/CA-SK/19	Biv	17-0773a/CA-AB/17
			Biv	19-0981/CA-SK/19
6	Biv	CC_CkAstV/US/2014	Biv	14-1235d/CA-AB/14,
			Bii	GA2011/US/2011
			Bii	4175/US/2011
7	Bii	GA2011/US/2011	Bii	4175/US/2011,
			Biv	19-0981/CA-SK/19,
			Biv	CC_CkAstV/US/2014,
			Biii	ANAND/IN/2016
			Biv	18-0942/CA-SK/18

## Supplementary Materials

The following are available online at https://www.mdpi.com/1999-4915/12/10/1096/s1, Table S1: Genome Sizes of complete CAstV Sequences; Figure S1: Amino acid ML phylogenetic tree of ORF1a; Figure S2: Amino acid ML phylogenetic tree of ORF1b; Figure S3: Nucleotide ML phylogenetic analyses on CAstV on each of the recombination events described on Table 2; Figure S4: Amino acid ML phylogenetic tree of a total of 83 partial ORF2 CAstV sequences; Figure S5: Percentage heat map of amino acid identity obtained from ML phylogenetic tree described on Figure S4.
