# Supplementary material for: Chicken Astrovirus (CAstV) Molecular Studies Reveal Evidence of Multiple Past Recombination Events in Sequences Originated from Clinical Samples of White Chick Syndrome (WCS) in Western Canada
Source: Viruses. 2020 Sep 28;12(10):1096. doi: 10.3390/v12101096 (PMC7600043; doi:10.3390/v12101096)
Supplement: Supplementary file 1 [file viruses-12-01096-s001.pdf]

Supplement Table 1. Genome sizes of complete CAstV sequences

ID	ORF1a	ORF1b	ORF2	Genome size
14-1235a-AB	3,423	1,560	2,217	7,501
14-1235b-AB	3,423	1,560	2,217	7,501
14-1235c-AB	3,423	1,560	2,217	7,459
14-1235d-AB	3,423	1,560	2,217	7,459
15-1262a-AB	3,423	1,560	2,217	7,506
15-1262b-AB	3,423	1,560	2,217	7,495
15-1262c-AB	3,423	1,560	2,217	7,504
15-1262d-AB	3,423	1,560	2,217	7,507
17-0773a-AB	3,420	1,560	2,217	7,452
17-0773b-AB	3,420	1,560	2,217	7,490
17-0823-AB	3,420	1,560	2,217	7,502
18-0942-SK	3,420	1,560	2,217	7,490
19-0935-SK	3,420	1,560	2,217	7,481
19-0981-SK	3,420	1,560	2,217	7,452

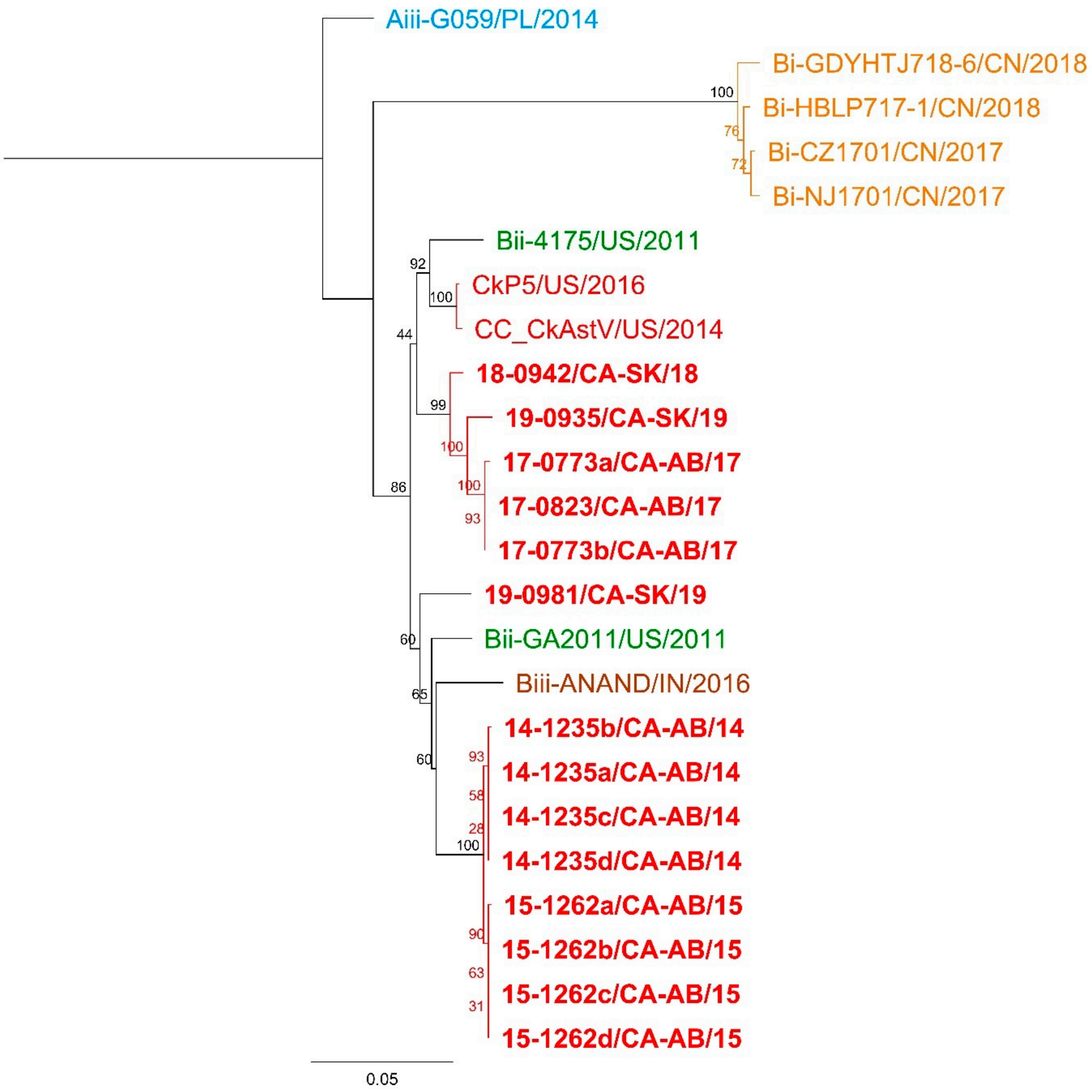

Supplement Fig 1. Amino acid ML phylogenetic tree of ORF1a CAstV sequences. Different colors indicate different genotypes (i.e. Aiii, Bi, Bii, Biii, and Biv in red) according to ORF2 analysis described in Smyth2017 [1]. The included sequences are described in Table 2. Canadian sequences are in bold.

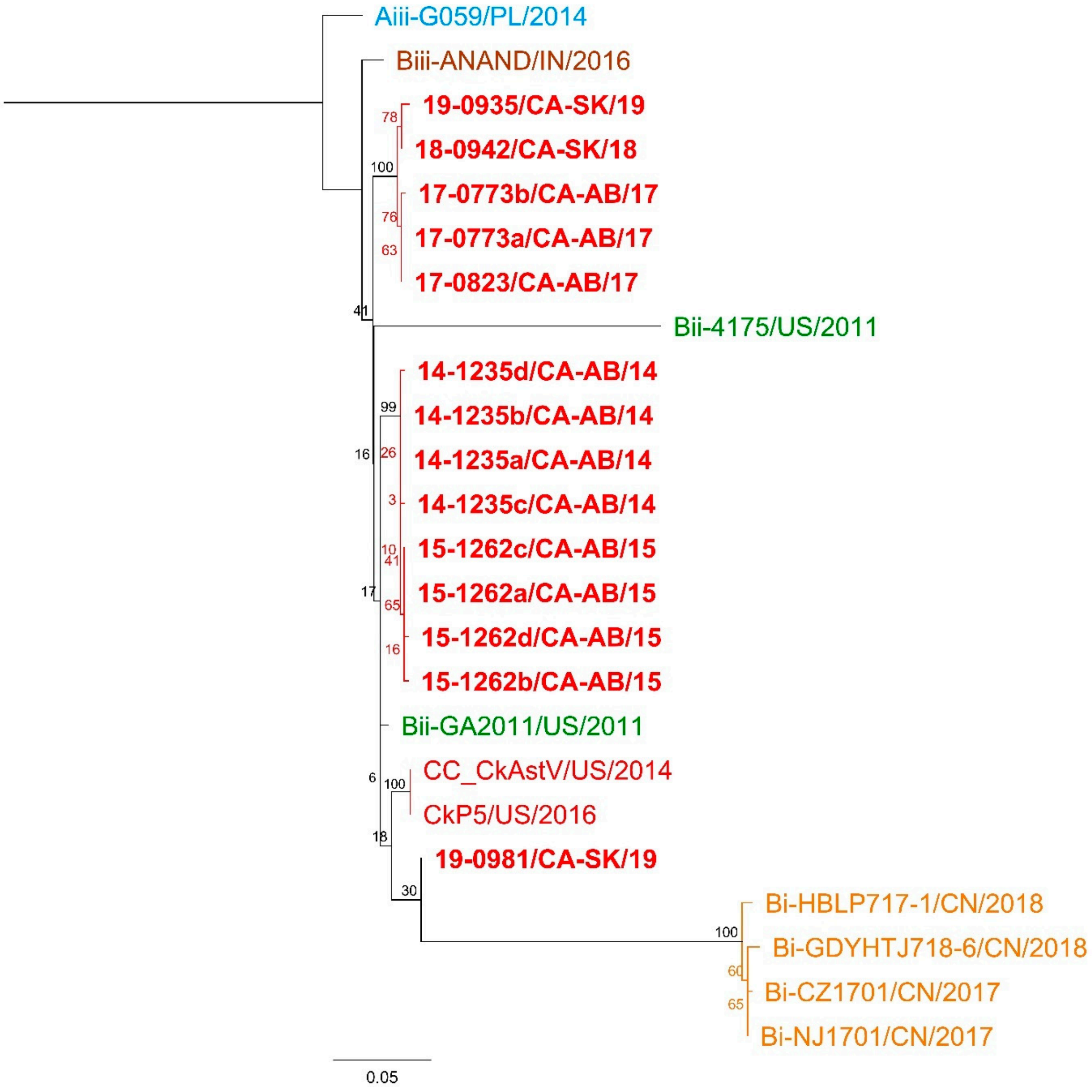

Supplement Fig 2. Amino acid ML phylogenetic tree of ORF1b CAstV sequences. Different colors indicate different genotypes (i.e. Aiii, Bi, Bii, Biii, and Biv in red) according to ORF2 analysis described in Smyth2017 [1]. The included sequences are described in Table 2 . Canadian sequences are in bold.

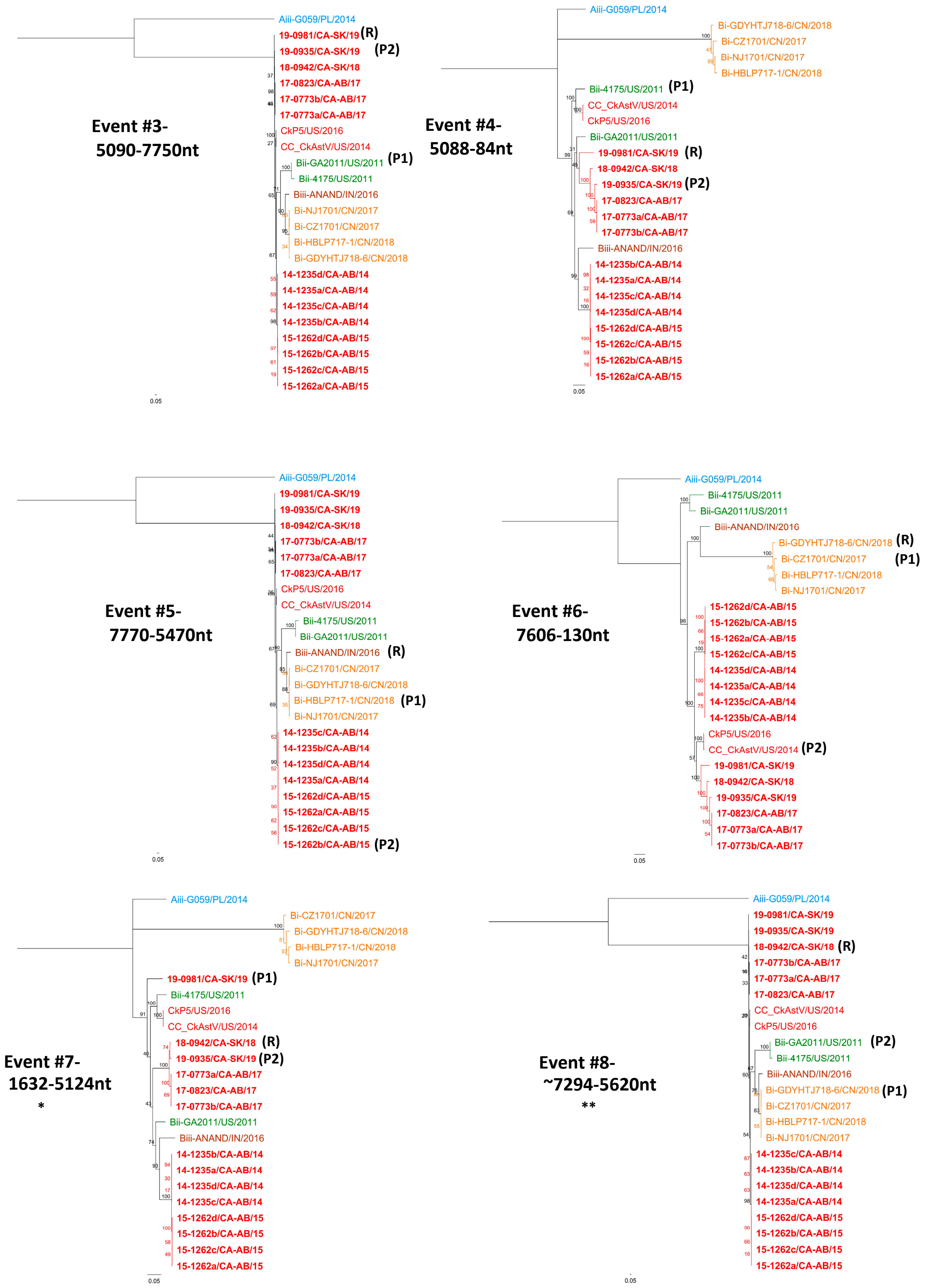
Event #3-
5090-7750nt
Aiii-G059/PL/2014
19-0981/CA-SK/19 (R)
19-0935/CA-SK/19 (P2)
18-0942/CA-SK/18
17-0823/CA-AB/17
17-0773b/CA-AB/17
17-0773a/CA-AB/17
CkP5/US/2016
CC_CkAstV/US/2014
Bii-GA2011/US/2011 (P1)
Bii-4175/US/2011
Biii-ANAND/IN/2016
Bi-NJ1701/CN/2017
Bi-CZ1701/CN/2017
Bi-HBLP717-1/CN/2018
Bi-GDYHTJ718-6/CN/2018
14-1235d/CA-AB/14
14-1235a/CA-AB/14
14-1235c/CA-AB/14
14-1235b/CA-AB/14
15-1262d/CA-AB/15
15-1262b/CA-AB/15
15-1262c/CA-AB/15
15-1262a/CA-AB/15
0.05
Event #4-
5088-84nt
Aiii-G059/PL/2014
Bi-GDYHTJ718-6/CN/2018
Bi-CZ1701/CN/2017
Bi-NJ1701/CN/2017
Bi-HBLP717-1/CN/2018
Bii-4175/US/2011 (P1)
CC_CkAstV/US/2014
CkP5/US/2016
Bii-GA2011/US/2011
19-0981/CA-SK/19 (R)
18-0942/CA-SK/18
19-0935/CA-SK/19 (P2)
17-0823/CA-AB/17
17-0773a/CA-AB/17
17-0773b/CA-AB/17
Biii-ANAND/IN/2016
14-1235b/CA-AB/14
14-1235a/CA-AB/14
14-1235c/CA-AB/14
14-1235d/CA-AB/14
15-1262d/CA-AB/15
15-1262c/CA-AB/15
15-1262b/CA-AB/15
15-1262a/CA-AB/15
0.05
Event #5-
7770-5470nt
Aiii-G059/PL/2014
19-0981/CA-SK/19
19-0935/CA-SK/19
18-0942/CA-SK/18
17-0773b/CA-AB/17
17-0773a/CA-AB/17
17-0823/CA-AB/17
CkP5/US/2016
CC_CkAstV/US/2014
Bii-4175/US/2011
Bii-GA2011/US/2011
Biii-ANAND/IN/2016 (R)
Bi-CZ1701/CN/2017
Bi-GDYHTJ718-6/CN/2018
Bi-HBLP717-1/CN/2018 (P1)
Bi-NJ1701/CN/2017
14-1235c/CA-AB/14
14-1235b/CA-AB/14
14-1235d/CA-AB/14
14-1235a/CA-AB/14
15-1262d/CA-AB/15
15-1262a/CA-AB/15
15-1262c/CA-AB/15
15-1262b/CA-AB/15 (P2)
0.05
Event #6-
7606-130nt
Aiii-G059/PL/2014
Bii-4175/US/2011
Bii-GA2011/US/2011
Biii-ANAND/IN/2016
Bi-GDYHTJ718-6/CN/2018 (R)
Bi-CZ1701/CN/2017 (P1)
Bi-HBLP717-1/CN/2018
Bi-NJ1701/CN/2017
15-1262d/CA-AB/15
15-1262b/CA-AB/15
15-1262a/CA-AB/15
15-1262c/CA-AB/15
14-1235d/CA-AB/14
14-1235a/CA-AB/14
14-1235c/CA-AB/14
14-1235b/CA-AB/14
CkP5/US/2016
CC_CkAstV/US/2014 (P2)
19-0981/CA-SK/19
18-0942/CA-SK/18
19-0935/CA-SK/19
17-0823/CA-AB/17
17-0773a/CA-AB/17
17-0773b/CA-AB/17
0.05
Event #7-
1632-5124nt
*
Aiii-G059/PL/2014
Bi-CZ1701/CN/2017
Bi-GDYHTJ718-6/CN/2018
Bi-HBLP717-1/CN/2018
Bi-NJ1701/CN/2017
19-0981/CA-SK/19 (P1)
Bii-4175/US/2011
CkP5/US/2016
CC_CkAstV/US/2014
18-0942/CA-SK/18 (R)
19-0935/CA-SK/19 (P2)
17-0773a/CA-AB/17
17-0823/CA-AB/17
17-0773b/CA-AB/17
Bii-GA2011/US/2011
Biii-ANAND/IN/2016
14-1235b/CA-AB/14
14-1235a/CA-AB/14
14-1235d/CA-AB/14
14-1235c/CA-AB/14
15-1262d/CA-AB/15
15-1262b/CA-AB/15
15-1262c/CA-AB/15
15-1262a/CA-AB/15
0.05
Event #8-
~7294-5620nt
**
Aiii-G059/PL/2014
19-0981/CA-SK/19
19-0935/CA-SK/19
18-0942/CA-SK/18 (R)
17-0773b/CA-AB/17
17-0773a/CA-AB/17
17-0823/CA-AB/17
CC_CkAstV/US/2014
CkP5/US/2016
Bii-GA2011/US/2011 (P2)
Bii-4175/US/2011
Biii-ANAND/IN/2016
Bi-GDYHTJ718-6/CN/2018 (P1)
Bi-CZ1701/CN/2017
Bi-HBLP717-1/CN/2018
Bi-NJ1701/CN/2017
14-1235c/CA-AB/14
14-1235b/CA-AB/14
14-1235d/CA-AB/14
14-1235a/CA-AB/14
15-1262d/CA-AB/15
15-1262b/CA-AB/15
15-1262c/CA-AB/15
15-1262a/CA-AB/15
0.05

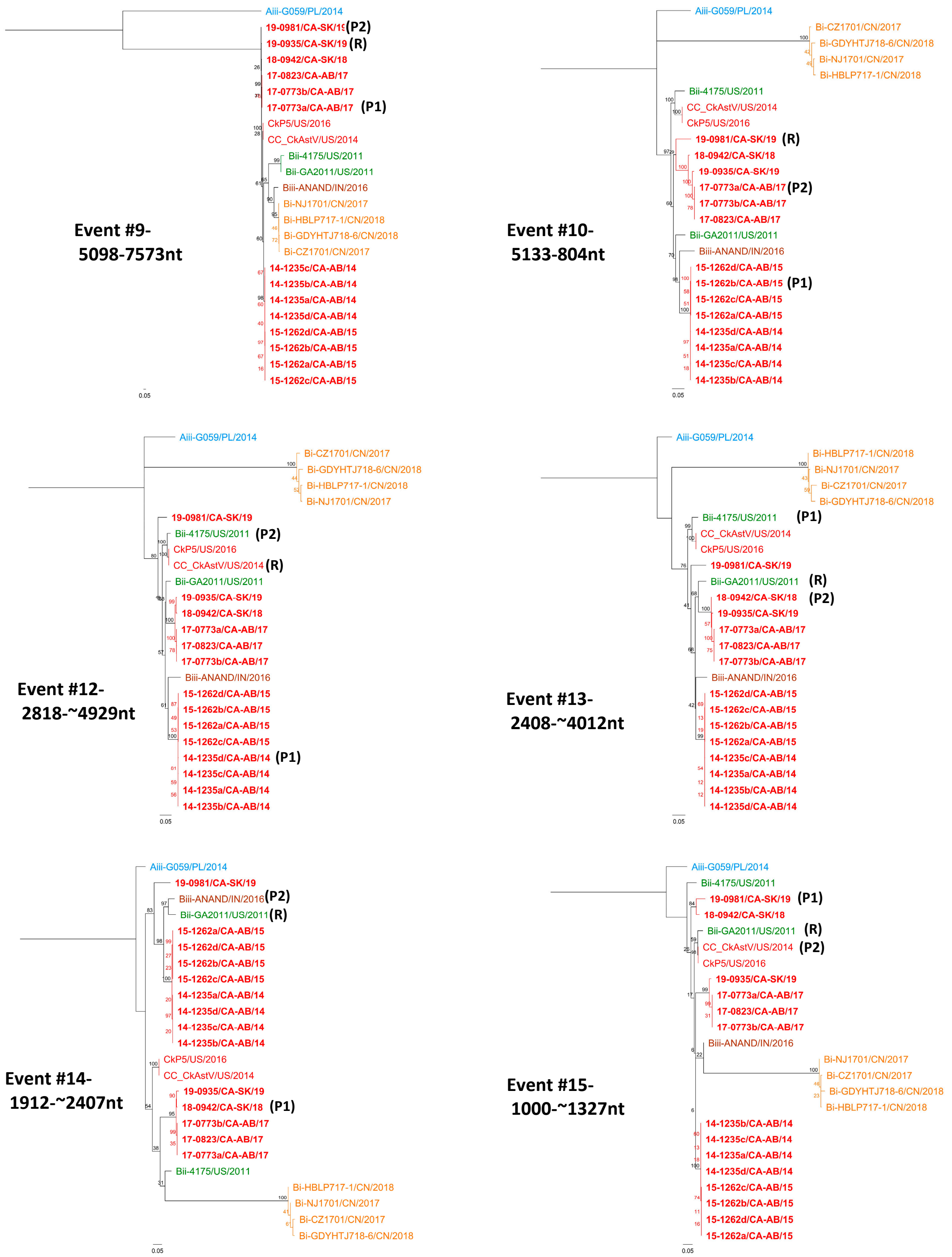

Supplement Fig 3. Nucleotide ML phylogenetic analyses of CAstV on each of the recombination events described on Table 2. Recombination events with genome positions as follows: Event#3-5090-7750nt; Event#4-5088-84nt; Event#5-7770-5470nt; Event#6-7606-130nt; Event#7-1632-5124nt; Event#8-~7294-5620nt; Event#9-5098-7573nt; Event #10-5133-804nt; Event#12-2818-~4929nt; Event#13-2408-~4012nt; Event#14-1912-~2407nt; and Event#15-1000-~1327nt. Different colors indicate different genotypes according to ORF2 analysis described in Smyth et al 2017 (i.e. Aiii, Bi, Bii, Biii, and Biv in red) [1]. The trees were built using

RAxML v 8.2.11 plugin of Geneious v.10.2.6. on an alignment obtained by Clustal Omega v 1.2.2. (R) Recombinant; (P1) Major Parent; (P2) Minor Parent. * Beginning breakpoint outside of confidence interval. ** Recombination signal may be attributable to a process other than recombination; ~ Unknown breaking point, approximate location noted.

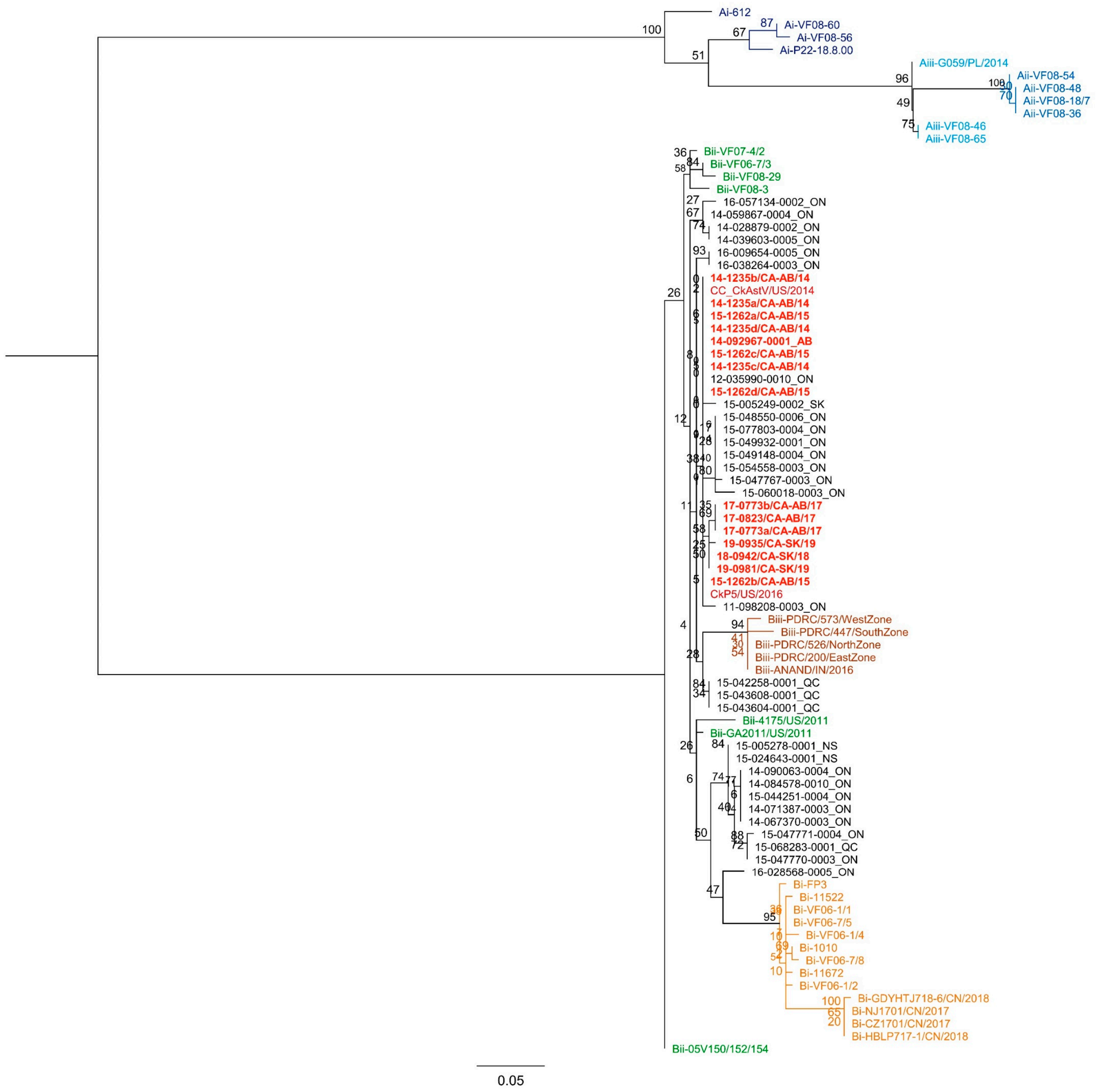

Supplement Fig 4. Amino acid ML phylogenetic tree of a total of 83 partial ORF2 CAstV sequences (aa positions 1-~214aa). Different colors indicate different genotypes (i.e. Ai, Aii, Aiii, Bi, Bii, Biii, and Biv in red), sequences in color black were initially classified as Bii, but classification is unclear. The included sequences are described in Table 2 and at Long et al 2018 [2]. Sequences obtained in this study are in bold.

	Bi-CZ1701/CN/	.P717-1/CN	J1701/CN/	HTJ718-6/C	3i-VF06-1/4	Bi-FP3	Bi-11672	Bi-11522	3i-VF06-1/	3i-VF06-7/	Bi-1010	3i-VF06-1/	3i-VF06-7/	RC/447/Sou	RC/573/We	RC/200/Ea	NAND/IN,	[illegible]
Bi-CZ1701/CN/2017		100	100	99.5	94.9	95.3	95.3	95.3	95.3	94.9	95.3	95.8	95.8	87.4	90.2	89.7	89.7	[illegible]
Bi-HBLP717-1/CN/2018	100		100	99.5	94.9	95.3	95.3	95.3	95.3	94.9	95.3	95.8	95.8	87.4	90.2	89.7	89.7	[illegible]
Bi-NJ1701/CN/2017	100	100		99.5	94.9	95.3	95.3	95.3	95.3	94.9	95.3	95.8	95.8	87.4	90.2	89.7	89.7	[illegible]
Bi-GDYHTJ718-6/CN/2018	99.5	99.5	99.5		94.4	94.9	94.9	94.9	94.9	94.4	94.9	95.3	95.3	86.9	89.7	89.3	89.3	[illegible]
Bi-VF06-1/4	94.9	94.9	94.9	94.4		98.1	98.6	98.6	98.6	98.1	98.6	99.1	99.1	89.7	92.1	92.1	92.1	[illegible]
Bi-FP3	95.3	95.3	95.3	94.9	98.1		98.6	98.6	98.6	98.1	98.6	99.1	99.1	90.2	92.5	92.5	92.5	[illegible]
Bi-11672	95.3	95.3	95.3	94.9	98.6	98.6		99.1	99.1	98.6	99.1	99.5	99.5	89.7	92.1	92.1	92.1	[illegible]
Bi-11522	95.3	95.3	95.3	94.9	98.6	98.6	99.1		99.1	98.6	99.1	99.5	99.5	89.7	92.1	92.1	92.1	[illegible]
Bi-VF06-1/2	95.3	95.3	95.3	94.9	98.6	98.6	99.1	99.1		98.6	99.1	99.5	99.5	89.7	92.1	92.1	92.1	[illegible]
Bi-VF06-7/8	94.9	94.9	94.9	94.4	98.1	98.1	98.6	98.6	98.6		99.5	99.1	99.1	89.3	91.6	91.6	91.6	[illegible]
Bi-1010	95.3	95.3	95.3	94.9	98.6	98.6	99.1	99.1	99.1	99.5		99.5	99.5	89.7	92.1	92.1	92.1	[illegible]
Bi-VF06-1/1	95.8	95.8	95.8	95.3	99.1	99.1	99.5	99.5	99.5	99.1	99.5		100	90.2	92.5	92.5	92.5	[illegible]
Bi-VF06-7/5	95.8	95.8	95.8	95.3	99.1	99.1	99.5	99.5	99.5	99.1	99.5	100		90.2	92.5	92.5	92.5	[illegible]
Biii-PDRC/447/SouthZone	87.4	87.4	87.4	86.9	89.7	90.2	89.7	89.7	89.7	89.3	89.7	90.2	90.2		96.7	97.7	97.7	[illegible]
Biii-PDRC/573/WestZone	90.2	90.2	90.2	89.7	92.1	92.5	92.1	92.1	92.1	91.6	92.1	92.5	92.5	96.7		99.1	99.1	[illegible]
Biii-PDRC/200/EastZone	89.7	89.7	89.7	89.3	92.1	92.5	92.1	92.1	92.1	91.6	92.1	92.5	92.5	97.7	99.1		100	[illegible]
Biii-ANAND/IN/2016	89.7	89.7	89.7	89.3	92.1	92.5	92.1	92.1	92.1	91.6	92.1	92.5	92.5	97.7	99.1	100		[illegible]
Biii-PDRC/526/NorthZone	89.7	89.7	89.7	89.3	92.1	92.5	92.1	92.1	92.1	91.6	92.1	92.5	92.5	97.7	99.1	100	100	[illegible]
16-028568-0005_ON	91.6	91.6	91.6	91.1	93	93.9	93.5	93.5	93.5	93	93.5	93.9	93.9	90.7	92.1	93	93	[illegible]
15-068283-0001_QC	92.1	92.1	92.1	92.5	93.5	93.9	93.5	93.5	93.5	93	93.5	93.9	93.9	90.2	91.6	92.5	92.5	[illegible]
15-047770-0003_ON	92.1	92.1	92.1	92.5	93.5	93.9	93.5	93.5	93.5	93	93.5	93.9	93.9	90.2	91.6	92.5	92.5	[illegible]
15-047771-0004_ON	91.6	91.6	91.6	92.1	93	93.5	93	93	93	92.5	93	93.5	93.5	90.2	91.6	92.5	92.5	[illegible]
15-005278-0001_NS	91.6	91.6	91.6	92.1	93.5	93.9	93.5	93.5	93.5	93	93.5	93.9	93.9	90.7	92.1	93	93	[illegible]
15-024643-0001_NS	91.6	91.6	91.6	92.1	93.5	93.9	93.5	93.5	93.5	93	93.5	93.9	93.9	90.7	92.1	93	93	[illegible]
[illegible]	[illegible]	[illegible]	[illegible]	[illegible]	[illegible]	[illegible]	[illegible]	[illegible]	[illegible]	[illegible]	[illegible]	[illegible]	[illegible]	[illegible]	[illegible]	[illegible]	[illegible]	[illegible]

Supplement Fig 5. Percentage of amino acid identity obtained from ML phylogenetic tree described on Supplement Fig 4.

References

1. Smyth, V. J., A Review of the Strain Diversity and Pathogenesis of Chicken Astrovirus. *Viruses* **2017,** 9, (2).
2. Long, K. E.; Ouckama, R. M.; Weisz, A.; Brash, M. L.; Ojkic, D., White Chick Syndrome Associated with Chicken Astrovirus in Ontario, Canada. *Avian diseases* **2018,** 62, (2), 247-258.
